# Electroacupuncture reduces chronic fibromyalgia pain through attenuation of transient receptor potential vanilloid 1 signaling pathway in mouse brains

**DOI:** 10.22038/ijbms.2020.39708.9408

**Published:** 2020-07

**Authors:** Chia-Ming Yen, Ching-Liang Hsieh, Yi-Wen Lin

**Affiliations:** 1 College of Chinese Medicine, Graduate Institute of Acupuncture Science, China Medical University, Taichung 40402, Taiwan; 2 Department of Anesthesiology, Taichung Tzu Chi Hospital, Buddhist Tzu Chi Medical Foundation; 3 College of Chinese Medicine, Graduate Institute of Integrated Medicine, China Medical University, Taichung 40402, Taiwan; 4 Chinese Medicine Research Center, China Medical University, Taichung 40402, Taiwan

**Keywords:** Dorsal root ganglion, Electroacupuncture, Fibromyalgia pain, pERK, Spinal cord, TRPV1

## Abstract

**Objective(s):**

Fibromyalgia pain is a mysterious clinical pain syndrome, characterized by inflammation in the brain, whose molecular mechanisms are still unknown. Females are more commonly affected by fibromyalgia, exhibiting symptoms such as widespread mechanical pain, immune dysfunction, sleep disturbances, and poor quality of life. Electroacupuncture (EA) has been used to relieve several types of pain, including fibromyalgia pain.

**Materials and Methods:**

In the present study, we used dual injections of acidic saline into the gastrocnemius muscle to initiate a neural activation that resulted in fibromyalgia pain in mice. We used the Von Frey test to measure mechanical hyperalgesia and Western blot to measure protein levels.

**Results:**

Results indicated that mechanical hyperalgesia can be induced in mice for 4 weeks, suggesting the induction of chronic fibromyalgia (CFM). Furthermore, continuous EA treatment reliably attenuated the mechanical hyperalgesia, but not in the sham control group. Results also suggested that the mechanical hyperalgesia can be prevented in mice with TRPV1 gene deletion. Mice with CFM showed increased expressions of TRPV1, Nav1.7, and Nav1.8 in the dorsal root ganglion (DRG) and the spinal cord (SC). The expression of TRPV1-associated molecules such as pPKA, pERK, and pCREB was also increased in the thalamus and somatosensory cortex (SSC) of the mice. All the aforementioned mechanisms were reversed by EA treatment and TRPV1 gene deletion.

**Conclusion:**

Altogether, our results implied significant mechanisms of CFM and EA-analgesia that involve the regulation of the TRPV1 signaling pathway. These findings may be relevant to the evaluation and treatment of CFM.

## Introduction

Chronic pain is a major healthcare issue across the world, with an estimated 20% of adults suffering from chronic, moderate-to-severe pain. Chronic pain is exceedingly rampant and associated with one of the highest healthcare costs worldwide (1). Studies using murine models of chronic pain have demonstrated evidence that can attest to the reorganization of the central nervous system, primarily emphasizing alterations of nociceptors and involvement of the spinal cord circuit (2, 3). Chronic pain not only correlates with painful processes but is also associated with central brain cognition and function (4). During the developmental process of pain signaling, harmful stimuli induce a response at the site of injury, initiating the release of inflammatory mediators such as histamine, bradykinin, serotonin, prostaglandins, ATP, protons, NGF, lipids, substance P, CGRP, and neurotransmitters. These stimuli can further activate representative receptors, which sensitize or activate peripheral nerve terminals for pain transduction (5).

Fibromyalgia (FM) is a disabling, chronic disease that requires long-term disease management. It manifests in the form of chronic, widespread sensations of nociception. This pathogenesis itself is puzzling because of the limited research available to identify the etiological nature. The prevalence of this debilitating condition has been approximated at 2%–8% among the general population (6). Patients feel spreading pain that is accompanied by fatigue, depression, memory problems, anxiety, sleep disturbances, and headaches. These resultant symptoms play an important role in the functional ability of individuals on a day-to-day basis. Chronic, long-term illness of the body can debilitate an individual, which emphasizes the importance of understanding the underlying mechanism to develop effective treatment solutions (7, 8). Several studies have used an animal model of FM induced through repeated acidic saline injections into the gastrocnemius muscle (GM). This type of chronic muscle pain model produces symptoms that are similar to those recorded in clinical patients, with long-lasting spreading mechanical hyperalgesia, fatigue, sympathetic predominance, and altered central sensitization being the primary features (8, 9). These FM animals are sensitive to antidepressants and anticonvulsants, but not to nonsteroidal anti-inflammatory drugs (NSAIDS) (10). Yang *et al*. demonstrated that acupuncture therapy for FM was superior to drugs. They found that acupuncture combined with drugs and exercise significantly increased the pain thresholds (11). Similarly, researchers suggested that acupuncture produced therapeutic effects that resulted in immediate pain reduction in patients with FM (12).

TRPV1, also known as vanilloid receptor 1 or receptor for capsaicin, which is the main ingredient in hot chili peppers, was first confirmed as an activator. It is a Ca^2+^-permeable ion channel that can be activated by capsaicin, RTX, acidic solutions (pH<6), high temperatures (>43 ^°^C), and arachidonic acid (13, 14). TRPV1 is also involved in the perception of inflammatory and thermal pain, especially pain caused due to the heat of more than 43 °C. The activation of TRPV1 can further initiate calcium influx for neuronal depolarization (15). Deletion of the TRPV1 gene results in an insensitivity to high-temperature stimulation, radial heat, and hot-plate tests. The expression of inflammatory mediators implicated in thermal hyperalgesia was also found to be reduced in TRPV1-null mice, suggesting its important role in thermal pain (16). Another study using an inflammatory pain model reported that subcutaneous or intrathecal injection of the TRPV1 antagonist, capsazepine (CPZ), reduced thermal hyperalgesia (17).

Acupuncture is a well-documented therapeutic strategy used for pain management, which originated in Asia about 3,000 years ago. Recent evidence demonstrates that acupuncture increases the release of adenosine at peripheral sites (18). It has been suggested that acupuncture can reliably increase the levels of both ATP and adenosine at the peripheral sites to attenuate inflammatory and neuropathic pain (18). Several studies have suggested that electroacupuncture (EA) increases the concentrations of endogenous opiates (19), serotonin (20), and adenosine (18), which are used to treat pain. Besides, EA can be used to attenuate stroke-induced dementia (21), Parkinson’s disease (22), epilepsy (23), and pain (9, 16, 24). In the present study, we intended to investigate the effect and mechanisms of EA on chronic FM pain and its relationship with TRPV1 and related downstream molecules. We hypothesized that TRPV1 and related molecules are involved in chronic FM pain and that EA treatment can attenuate FM pain through TRPV1 and related molecules.

## Materials and Methods

***Animals***

C57BL/6 female mice aged 8–12 weeks, which were purchased from BioLASCO Co. Ltd, Taipei, Taiwan, were used for all experiments. Mice were randomly assigned to five groups (n = 8 per group): (1) Normal, (2) CFM, (3) CFM+2Hz EA, (4) FM+sham EA, and (5) TRPV1^-/-^ groups. The sample size required for an alpha of 0.05 and a power of 80% was 8 animals per group. After arrival, mice were housed in a 12/12 hr light/dark cycle room with water and food available *ad libitum*. All procedures were approved by the Institute of Animal Care and Use Committee of China Medical University (No. 2018-110), which were conducted in accordance with the Guide for the Use of Laboratory Animals provided by the National Research Council and the ethical guidelines of the International Association for the Study of Pain. The number of animals used and their suffering were minimized.

***FM induction and mechanical hyperalgesia measurement***

All mice except controls received a 20 μl injection of acidic saline (pH 4.0) into the right gastrocnemius muscle (GM) under isoflurane (1%) anesthesia on day 0. The second acidic saline injection was administered on day 1 to establish the FM mouse model. The acidic saline was prepared in 10 mM 2-[N-morpholino]ethanesulfonic acid and further adjusted to pH 4.0 with 1 N NaOH. Mechanical sensitivity was tested after the first acidic saline injection. Mechanical sensitivity was measured by testing the strength of responses to stimulation with three applications of electronic Von Frey filaments (North Coast Medical, Gilroy, CA, USA). Mice were placed on a metal mesh and adapted to the new environment for at least 30 min before testing.

***Electroacupuncture treatment***

Acupuncture needles (0.5 inch, 32 G, YU KUANG, Taiwan) were inserted into the muscle layer at a depth of 3–5 mm in the ST36 acupoint under 1% isoflurane anesthetization. In the sham group, the needle was inserted into the ST36 acupoint without any rotation or twisting. The location of ST36 is approximately 3 mm below and 1–2 mm lateral to the midpoint of the knee in mice. To ensure an insertion depth of 3 mm, a piece of tape was stuck to the needle, leaving only space for manipulation and a needle tip of 3 mm. EA treatment was applied using 2 Hz stimulation by delivering electrical stimulation with a Trio 300 electrical stimulator (Grand Medical Instrument Co. Ltd). The electrical pulses were delivered at 100-μs square pulses of 1 mA for 15 min at 2 Hz. ST36 was chosen as it is commonly used in traditional Chinese medicine; the analgesic effects of EA at ST36 are well established (9, 16, 24). 

***Tissue sampling and Western blot analysis***

Lumbar dorsal root ganglion (DRG), spinal cord (SC), brain thalamus, and somatosensory cortex (SSC) were excised immediately to extract proteins. The total proteins were prepared by homogenizing the tissues in lysis buffer containing 50 mM Tris-HCl (pH 7.4), 250 mM NaCl, 1% NP-40, 5 mM EDTA, 50 mM NaF, 1 mM Na_3_VO_4_, 0.02% NaNO_3_, and 1× protease inhibitor cocktail (AMRESCO). The extracted proteins (30 μg per sample according to the BCA protein assay) were subjected to 8% SDS-Tris glycine gel electrophoresis and transferred to a PVDF membrane. The membrane was blocked with 5% non-fat milk in TBS-T buffer (10 mM Tris pH7.5, 100 mM NaCl, 0.1% Tween 20), incubated with the first antibody in TBS-T and 1% bovine serum albumin, and incubated for 1 hr at room temperature. A peroxidase-conjugated anti-rabbit antibody (1:5000) was used as the secondary antibody. The bands were visualized using an enhanced chemiluminescence substrate kit (PIERCE) with LAS-3000 Fujifilm (Fuji Photo Film Co. Ltd). If appropriate, the image intensities of specific bands were quantified using NIH ImageJ software (Bethesda, MD, USA). The protein ratios were obtained through dividing the target protein intensities by the intensity of α-tubulin in the same sample. The calculated ratios were then adjusted by dividing the ratios from the same comparison group relative to the control.

***Statistical analysis***

All of the data were expressed as the mean±standard error. Significant differences between the normal, CFM, CFM+2Hz EA, CFM+sham EA, and TRPV1^-/-^ groups were tested using ANOVA, followed by a *post hoc* Tukey’s test. *P*<0.05 was considered significantly different.

## Results

***EA attenuated chronic FM pain in mice***

**Figure 1:**
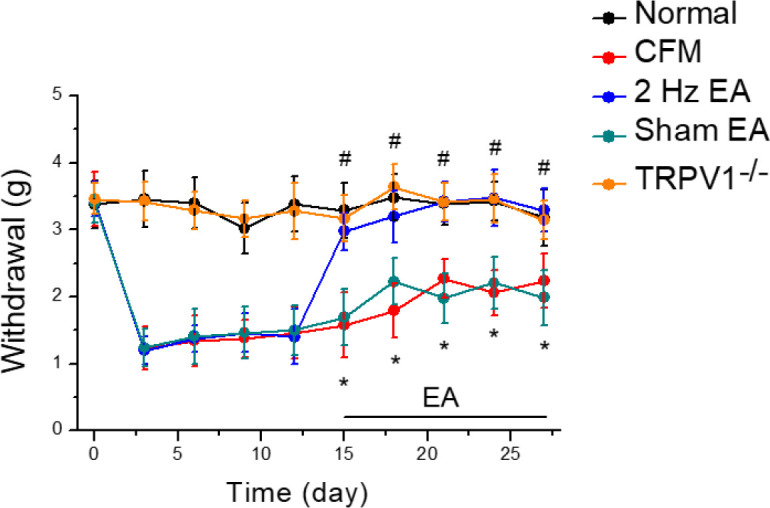
Mechanical pain thresholds in five groups of mice. Normal saline injection (Normal group, n=8), CFM (Acid saline-induced chronic FM pain), 2 Hz EA (Acid saline-induced chronic FM pain treated with 2 Hz EA), sham EA (Acid saline-induced FM pain treated with sham EA), and TRPV1-/- (Acid saline-induced FM pain in TRPV1-/- mice). *P<0.05 vs Normal group. #*P*<0.05 vs CFM group

 To evaluate the effect of EA on a chronic FM mice model, we injected acidic saline into mice GM. After the induction of FM, mechanical hyperalgesia was observed and maintained for 4 weeks (Figure 1, red circle, n=8). After 2 weeks of mechanical hyperalgesia, similar to clinical observation, EA treatment for 2 weeks significantly attenuated this phenomenon (Figure 1, blue circle, n=8). The reversal of CFM could not be obtained in the sham EA group (Figure 1, green circle, n=8). In addition, deletion of the TRPV1 gene resulted in a reduction of mechanical hyperalgesia, suggesting the crucial role of TRPV1 in the CFM mice model (Figure 1, orange circle, n=8).

***The expression of TRPV1, Nav1.7, and Nav1.8 was altered in the peripheral dorsal root ganglion and central SC of CFM mice***

**Figure 2:**
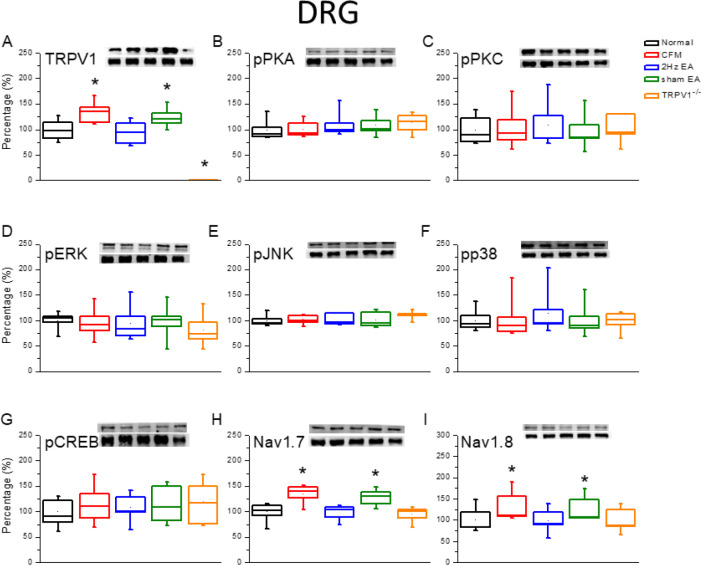
Expression levels of TRPV1-associated signaling pathways in the mice lumbar DRG. (A) TRPV1, (B) pPKA, (C) pPKC, (D) pERK, (E) JNK, (F) pp38, (G) pCREB, (H) nav1.7, and (I) Nav1.8 expression levels in Normal, CFM, CFM+2 Hz EA, CFM+sham EA, and TRPV1-/- mice (from left to right). Normal: normal mice; CFM: chronic fibromyalgia mice; 2 Hz EA: CFM+2 Hz EA. Sham EA: CFM+sham EA. TRPV1-/-: CFM+TRPV1-/-. *P<0.05 compared with the normal group. The Western blot bands at the top show the target protein. The lower bands are internal controls (β-actin or α-tubulin)

**Figure 3:**
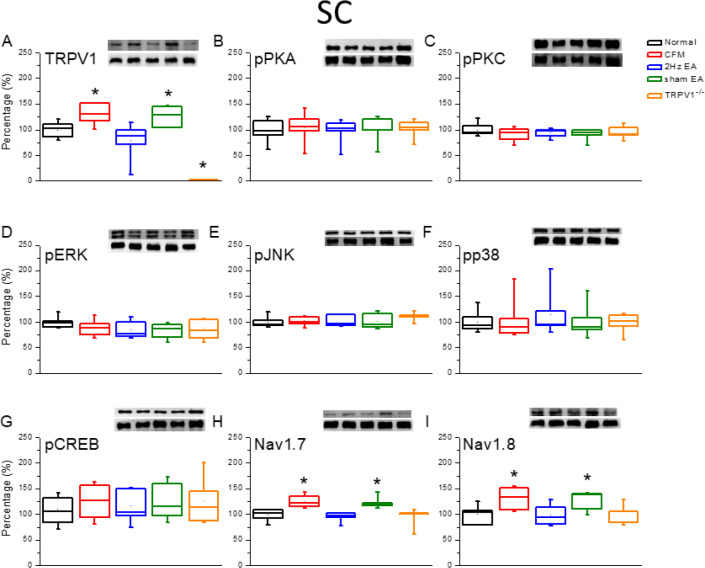
Expression levels of TRPV1-associated signaling pathways in the mice lumbar SC. (A) TRPV1, (B) pPKA, (C) pPKC, (D) pERK, (E) JNK, (F) pp38, (G) pCREB, (H) nav1.7, and (I) Nav1.8 expression levels in Normal, CFM, CFM+2 Hz EA, CFM+sham EA, and TRPV1-/- mice (from left to right). Normal: normal mice; CFM: chronic fibromyalgia mice; 2 Hz EA: CFM+2 Hz EA. Sham EA: CFM+sham EA. TRPV1-/-: CFM+TRPV1-/-. *P<0.05 compared with the normal group. The Western blot bands at the top show the target protein. The lower bands are internal controls (β-actin or α-tubulin)

The Western blotting technique was used to quantify TRPV1-related protein levels in the mice DRG. We observed that TRPV1 was expressed in the DRG of normal mice (Figure 2A, 100.1%±4.5%, *P*>0.05, n=6). The expression of TRPV1 was significantly increased in the DRG of chronic FM induced mice (Figure 2A, 120.5% ± 8.2%, *P*>0.05, n=6). In addition, potentiation of TRPV1 was reduced by continuous 2-Hz EA treatment (Figure 2A, 94.6%±5.0%, *P*<0.05, n=6), but not in the sham-operated EA group (Figure 2A, 114.2%±7.0%, *P*>0.05, n=6). The expression of TRPV1 protein was almost absent in the DRG of mice with TRPV1 gene deletion (Figure 2A, 3.5%±2.0%, *P*<0.05, n=6). We further assessed whether downstream molecules such as pPKA, pPKC, pERK, pJNK, pp38, and pCREB participated in the DRG of the CFM mice model. The levels of the abovementioned molecules remained unchanged in all groups, indicating that they were not involved in the peripheral DRG level at that time point (Figures 2B–G, *P*>0.05, n=6). We further observed that both Nav1.7 and Nav1.8 were potentiated in the DRG of CFM mice (Figures 2H and I, *P*<0.05, n=6). This increase in the protein levels was further reversed by EA treatment (Figures 2H and I, *P*<0.05, n=6), but not in the sham group (Figures 2H and I, *P*<0.05, n=6). Deletion of the TRPV1 gene also prevented the overexpression of Nav1.7 and Nav1.8 (Figures 2H and I, *P*<0.05, n=6). Similar results were obtained at the central SC level (Figure 3).

***Increased expression of the TRPV1 signaling pathway in the central thalamus and SSC***

**Figure 4:**
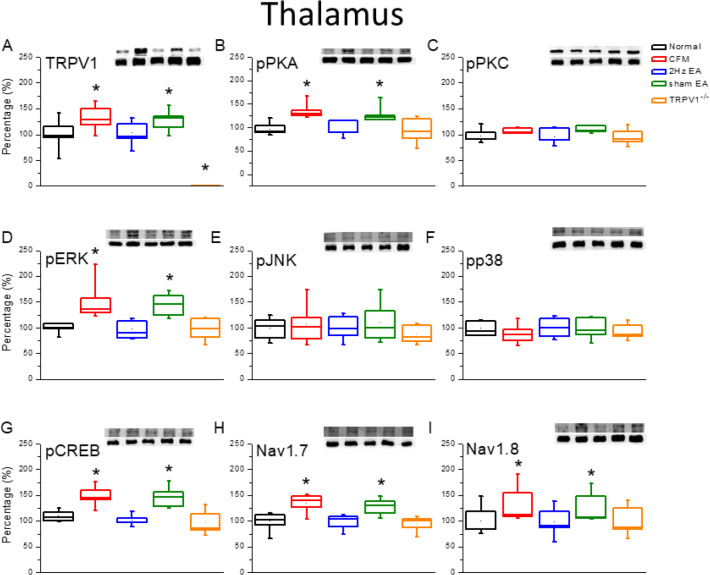
Expression levels of TRPV1-associated signaling pathways in the mice thalamus. (A) TRPV1, (B) pPKA, (C) pPKC, (D) pERK, (E) JNK, (F) pp38, (G) pCREB, (H) nav1.7, and (I) Nav1.8 expression levels in Normal, CFM, CFM+2 Hz EA, CFM+sham EA, and TRPV1-/- mice (from left to right). Normal: normal mice; CFM: chronic fibromyalgia mice; 2 Hz EA: CFM+2 Hz EA. Sham EA: CFM+sham EA. TRPV1-/-: CFM+TRPV1-/-. *P<0.05 compared with the normal group. The Western blot bands at the top show the target protein. The lower bands are internal controls (β-actin or α-tubulin)

We further determined whether the TRPV1-related signaling pathway was involved in the central thalamus to delineate its role in central sensitization. The results indicated that TRPV1 was expressed in the mice thalamus, with further increased expression in the CFM mice (Figure 4A, 100.1%±6.3% and 134.3%±9.2%, *P*<0.05, n=6). Furthermore, the potentiation was reversed by EA treatment (Figure 4A, 104.5%±12.6%, *P*<0.05, n=6), but not in the sham-operated group (Figure 4A, 129.0%±14.0%, *P*<0.05, n=6). Our results also suggested that pPKA expression was increased in the thalamus of CFM mice (Figure 4B, 133.2%±11.5%, *P*<0.05, n=6) and then reduced by treatment with 2-Hz EA (Figure 4B, 106.4%±9.6%, *P*<0.05, n=6), but not in the sham group (Figure 4B, 123.9%±13.5%, *P*>0.05, n = 6). The increase in pPKA protein level was also reversed in TRPV1^-/-^ mice (Figure 4B, 100.6%±12.0%, *P*<0.05, n=6). Furthermore, the results were not replicated for pPKC protein level, suggesting that it is not involved in this CFM mice model (Figure 4C, *P*>0.05, n=6). The expression of pERK was significantly increased in the thalamus of CFM mice (Figure 4D, 147.4%±17.5%, *P*<0.05, n=6) and then attenuated by EA treatment (Figure 4C, 107.5%±12.6%, *P*<0.05, n=6), but not in the sham group (Figure 4C, 131.7%±12.9%, *P*>0.05, n=6). Similar results were also obtained in the TRPV1^-/-^ group (Figure 4C, 109.3%±12.8%, *P*<0.05, n=6). However, these phenomena were not observed for pJNK (Figure 4C, *P*>0.05, n=6) and pp38 levels (Figure 4C, *P*>0.05, n= 6). The transcriptional factor pCREB was potentiated by CFM induction (Figure 4C, 121.3%±3.8%, *P*<0.05, n=6) and further reversed by EA treatment (Figure 4C, 94.8%±2.0%, *P*<0.05, n=6), but not in the sham group (Figure 4C, 120.7%±3.5%, *P*>0.05, n=6). In addition, deletion of the TRPV1 gene prevented this potentiation (Figure 4C, 91.6%±5.4%, *P*<0.05, n=6). Similar results were observed for the expression of Nav1.7 and nav1.8 proteins (Figures 4H–I, *P*<0.05, n=6).

**Figure 5:**
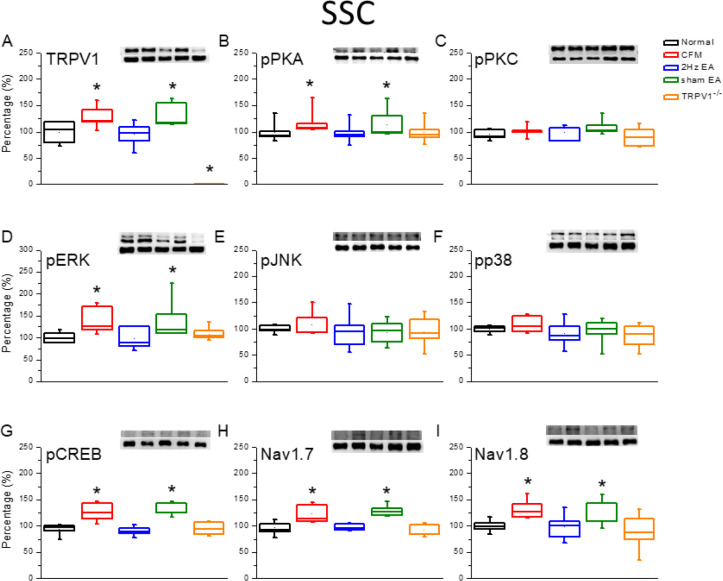
Expression levels of TRPV1-associated signaling pathways in the mice SSC. (A) TRPV1, (B) pPKA, (C) pPKC, (D) pERK, (E) JNK, (F) pp38, (G) pCREB, (H) nav1.7, and (I) Nav1.8 expression levels in Normal, CFM, CFM+2 Hz EA, CFM+sham EA, and TRPV1-/- mice (from left to right). Normal: normal mice; CFM: chronic fibromyalgia mice; 2 Hz EA: CFM+2 Hz EA. Sham EA: CFM+sham EA. TRPV1-/-: CFM+TRPV1-/-. *P<0.05 compared with the normal group. The Western blot bands at the top show the target protein. The lower bands are internal controls (β-actin or α-tubulin)

To determine whether CFM altered the levels of TRPV1-related molecules in SSC, suggesting central sensitization, we collected SSC after 4 weeks of FM induction. We observed that CFM induction significantly increased the expression of TRPV1 in mice SSC (Figure 5A, 124.4%±7.6%, *P*<0.05, n=6) compared with that in normal mice. Furthermore, EA treatment significantly reduced the levels of TRPV1 in the SSC (Figure 5A, 95.7%±8.0%, *P*<0.05, n=6). In addition, sham EA treatment did not change the TRPV1 protein level (Figure 5A, 126.6%±8.6%, *P*>0.05, n=6). Furthermore, there was no increase in the expression of TRPV1 in the gene-deleted mice (Figure 4C, 9.2%±1.4%, *P*<0.05, n=6). We further observed that, similar to the aforementioned results, pPKA, but not pPKC, was involved in CFM induction (Figures 5B and C, n=6). In addition, the levels of pERK rather than pJNK and pp38 were increased in SSC of CFM mice and were further reversed by EA treatment and in TRPV1^-/-^ mice, but not in sham mice (Figures 5D–F, n=6). The role of the transcription factor pCREB was also evident in this process (Figure 5G, *P*<0.05, n=6). Similar results were observed for the expression levels of Nav1.7 and Nav1.8 proteins (Figures 5H and I, *P*<0.05, n=6).

## Discussion

Fibromyalgia is a well-defined chronic pain disorder with key features such as widespread pain, sleep disturbances, cognitive dysfunction, and emotional distress. Patients always feel an unknown pain due to central and peripheral nerve sensitization. Accordingly, it has been recommended that fibromyalgia is a central hypersensitivity symptom. Recently, neurological and inflammatory mechanisms have been reported to be involved in the peripheral nerve system, spinal cord, and brain in fibromyalgia (25). Several studies have indicated that the levels of proinflammatory cytokines such as IL-1, IL-6, and IL-8 are elevated in the blood of patients with fibromyalgia (26-28). The involvement of the sympathetic nervous system and the dysfunction of the hypothalamic-pituitary-adrenal axis are often observed in patients with fibromyalgia (29). Extensive research suggests the critical role of TRPV1 in psychological disorders such as anxiety and depression (30). Marrone *et al*. reported that TRPV1 is a critical brain inflammation detector and a neuropathic pain biomarker in mice (31). The present study demonstrated that TRPV1 is elevated in the peripheral DRG of CFM mice and further in the central SC, thalamus, and SSC. The increase in activation of the TRPV1 signaling pathway was further reduced by EA treatment and gene deletion.

A recent study reported that there is significant comorbidity between pain, depression, or anxiety, which is approximately 20%–23%, the comorbidity of depression and chronic pain is scored with more severe pain (32). A strong association exists between fibromyalgia pain, sleep quality, and depression. A study found that poor sleep quality led to a worse situation in terms of pain sensation, psychological scores, and perceived quality of life (33). In addition, patients with stubborn neuropathic pain significantly develop concomitant depression or sleep disorders. Anticonvulsants and antidepressants are used for relieving neuropathic pain and sleep and psychological disorders, but these often initiate severe side effects (34). The present study showed that EA treatment or deletion of the TRPV1 gene reliably attenuated the CFM pain in mice.

Increasing evidence suggests that neural changes may occur in either the peripheral or the central nervous system. Researchers summarized the changes occurring in the thalamus, amygdala, basal ganglia, cerebellum, cingulate cortex, and putamen based on MRI analysis. Furthermore, increased activities in the prefrontal cortex, anterior cingulate cortex, thalamus, and SSC were detected in the fMRI measurements (35). A study reported that FM results in low selenium levels, high Ca^2+^ influx and reactive oxygen species (ROS) production, and acidic pH. Administration of selenium attenuated the increased TRPM2 and TRPV1 currents, pain intensity, and intracellular Ca^2+^ in the sciatic nerve and DRG (36). Maciel *et al*. reported that EA treatment at high or low frequency was effective in reducing hyperalgesia in the CFM model (37). 

Our recent study indicated that dual-acid saline injections successfully induced mechanical hyperalgesia via ASIC3, Nav1.7, and Nav1.8 signaling in peripheral DRG and central SC (9). The Nav1.8 protein level and sodium channel current were increased in mice DRG neurons after CFA-induced inflammatory pain (38, 39). An article indicated that visceral pain and referred hyperalgesia were reduced in Nav1.8-null mice (40). We previously suggested that EA attenuates CFA-induced inflammatory pain by attenuating Nav1.8 through TRPV1, opioid, and adenosine pathways in Mice (16). Intrathecal injection of Nav1.8 antisense dramatically blocked Nav1.8 currents and attenuated mechanical allodynia in a CFA inflammatory pain model (41). Our recent article suggests EA at acupoint ST36 reliably reduced mechanical hyperalgesia in acidic saline injection-induced FM mice. pPKA, pPI3K, and pERK signaling pathways were increased in the mice DRG and SC. In addiction ASIC3, Nav1.7, and Nav1.8 proteins were increased at day 8 after FM induction, and further reversed by EA (9). Our results showed that the levels of TRPV1, Nav1.7, and Nav1.8, which are reported as nociceptive channels, were increased in DRG. The TRPV1-related signaling pathway was dramatically increased in the brain thalamus and SSC, thus suggesting central sensitization.

## Conclusion

In the present study, we created a CFM mice model, which was maintained for 4 weeks with chronic widespread pain, using dual acidic saline injections. We demonstrated that continuous EA treatment significantly reduced mechanical hyperalgesia in CFM mice, but not in the sham-operated group. In addition, deletion of the TRPV1 gene also prevented mechanical hyperalgesia. Nociceptive channels such as TRPV1, Nav1.7, and Nav1.8 were simultaneously increased in DRG and SC of CFM mice. The levels of the TRPV1-associated signaling pathway molecules such as pPKA, pERK, and pCREB were all increased in the brain thalamus and SSC of CM mice. This increase in all the aforementioned molecule levels was attenuated by EA treatment and TRPV1 gene deletion. Our results suggest the significance of the EA clinical therapy, which may be crucial for pain management.
